# Neurologic Characteristics in Coronavirus Disease 2019 (COVID-19): A Systematic Review and Meta-Analysis

**DOI:** 10.3389/fneur.2020.00565

**Published:** 2020-05-29

**Authors:** Rizaldy Taslim Pinzon, Vincent Ongko Wijaya, Ranbebasa Bijak Buana, Abraham Al Jody, Patrick Nalla Nunsio

**Affiliations:** ^1^Faculty of Medicine, Duta Wacana Christian University, Yogyakarta, Indonesia; ^2^Bethesda Hospital, Yogyakarta, Indonesia

**Keywords:** COVID-19, symptoms, characteristics, neurologic, review, meta-analysis

## Abstract

**Importance:** Coronavirus disease 2019 (COVID-19) is a newly emerging infectious disease that has caused a global pandemic. The presenting symptoms are mainly respiratory symptom, yet studies have reported nervous system involvement in the disease. A systematic review and meta-analysis of these studies are required to understanding the neurologic characteristic of the disease and help physicians with early diagnosis and management.

**Objective:** To conduct a systematic review and meta-analysis on the neurologic characteristics in patients with COVID-19.

**Evidence Review:** Authors conducted a literature search through PubMed from January 1st, 2020 to April 8th, 2020. Furthermore, the authors added additional sources by reviewing related references. Studies presenting the neurologic features of COVID-19 patients in their data were included. Case reports and case series were also included in this review. The quality of the studies was assessed based on the Oxford Center for Evidence-Based Medicine guidelines. Selected studies were included in the meta-analysis of proportion and the heterogeneity test.

**Finding:** From 280 identified studies, 33 were eligible, with 7,559 participants included. Most of the included studies were from China (29 [88%]). Muscle injury or myalgia was the most common (19.2%, 95%CI 15.4–23.2%) neurologic symptom of COVID-19, followed by headache (10.9%, 95%CI 8.62–13.51%); dizziness (8.7%, 95%CI 5.02–13.43%); nausea with or without vomiting (4.6%, 95%CI 3.17–6.27%); concurrent cerebrovascular disease (4.4%, 95%CI 1.92–7.91%); and impaired consciousness (3.8%, 95%CI 0.16–12.04%). Underlying cerebrovascular disease was found in 8.5% (95%CI 4.5–13.5%) of the studies.

**Conclusion:** Neurologic findings vary from non-specific to specific symptoms in COVID-19 patients. Some severe symptoms or diseases can present in the later stage of the disease. Physicians should be aware of the presence of neurologic signs and symptoms as a chief complaint of COVID-19, in order to improve management and prevent a worsening outcome of the patients.

## Introduction

In December 2019, three patients with pneumonia were observed and linked to the outbreak of respiratory infection cases detected from Wuhan, China. Later on, the cause of pneumonia was found to be a viral infection known as novel coronavirus disease (COVID-19). In March 2020, the World Health Organization (WHO) declared COVID-19 as an emerging infectious disease caused by the virus SARS-CoV-2 (severe acute respiratory syndrome coronavirus 2) and declared a global pandemic. As of April 7, 2020, globally reported cases are 1.279.722 confirmed cases with more than 70.000 deaths (1, 2).

The disease's main presentations are usually similar to symptoms of upper respiratory tract infection such as fever, dry cough, and myalgia or malaise. In severe cases, manifestations of pneumonia such as shortness of breath (dyspnea), abnormal lung imaging findings, and acute respiratory distress syndrome (ARDS) can be found (3). Multiple studies have also reported the nervous system involvement in the disease. A retrospective study in China found that over 36.4% of hospitalized patients had neurologic symptoms and that these commonly present in severe patients (4, 5).

Studies also suggested that physicians should be aware of the other system involvement, including neurological events, to reduce mortality and morbidity rate in affected individuals. This review aims to provide a systematic report of the neurologic characteristics in patients with COVID-19 based on the latest reported studies.

## Methods

### Literature Searching

We performed a systematic literature review, followed by meta-analysis, of the available studies from one scientific database (PubMed), published from January 1st, 2020, to April 8th, 2020, using Preferred Reporting Items for Systematic Reviews and Meta-analyses (PRISMA) reporting guideline. The following inclusion criteria were: (1) original studies (e.g., randomized controlled trial studies, cohort studies, case-control studies, cross-sectional studies, case reports, and case-series) on patients with COVID-19; (2) Studies with a focus on clinical manifestations or symptoms in patients with COVID-19; and (3) The literature was restricted to English language articles only. We excluded the following studies: non-original articles, such as review articles including meta-analyses, letters, comments, or consensus documents (6).

We included clinical characteristics studies as long as they contained neurologic data of COVID-19. As the disease is an urgent topic and a newly emerging disease, we also included case reports or case series in the review.

Two co-authors (V.O.W and A.A) independently screened the titles in each study from the search results for eligibility. Each of the abstracts were examined when eligibility was not clear from the title. The search was performed by using terms “COVID-19” AND “characteristics,” as well as their derivations from the selected articles. We minimized our search keywords to expand our findings and to obtain more studies. We prioritized our aim to collect the neurological characteristics data of the disease. Finally, additional articles were added based on the bibliography of the articles retrieved through the outlined search strategy and were manually screened to refine this review.

### Data Extraction

Two independent reviewers (P.N.N and R.B.B) then assessed the text articles that passed the first screening process to ensure their eligibility and compliance with inclusion and exclusion criteria. Then, the reviewers identified every article bibliography, within each document that discussed the clinical characteristics in COVID-19, and specifically searched for the neurologic characteristics in the text, to be added into the additional records. When the reviewers could not reach consensus, the main author would assess the review relevance for a final decision.

The following data were recorded and tabulated from all reviewed articles: author names, study design, country location, study group, age, neurologic symptoms, key findings, and study limitations.

### Study Quality Assessment

We assessed the quality of each study using The Oxford Center for Evidence-Based Medicine Quality ratings. The ratings ranged from 1 to 5, with 1 representing properly powered and adequate randomized controlled trial (RCT) and 5 representing opinions and case reports (7).

### Analysis

We conducted the meta-analysis with prevalence estimates, that had been transformed using the Freeman-Tukey transformation (arcsine method), to calculate the weight proportion under the random-effects model. A pooled prevalence figure was calculated with 95% CI. The pooled prevalence of neurologic manifestations was estimated from the reported prevalence of eligible studies. Forest plots were generated, displaying prevalence for each study. The overall random-effects pooled estimate with its CI was reported. We limited the articles included in the meta-analysis to those manifestations that were present in more than one study and excluded the case reports (8).

The meta-analysis was performed using a random-effects model to account for heterogeneity. Heterogeneity between estimates was assessed using the *I*^2^ statistic, which describes the percentage of variation not because of sampling error across studies. An *I*^2^ value above 75% indicates high heterogeneity. Statistical significance was declared at *I*^2^ > 50% and *p* < 0.05. The analysis was done using MedCalc V.19.2.0 software (8).

## Results

### Methodological Quality of Included Studies

Most of the studies included were observational studies. Of the 33 included studies, 19 (58%) were cohort studies, 10 (30%) were retrospective case series or cross sectional studies, and four (12%) were case reports. Individual study quality ratings are presented in Supplementary Table 1.

### Study Results and Patient Characteristics

**Figure 1:**
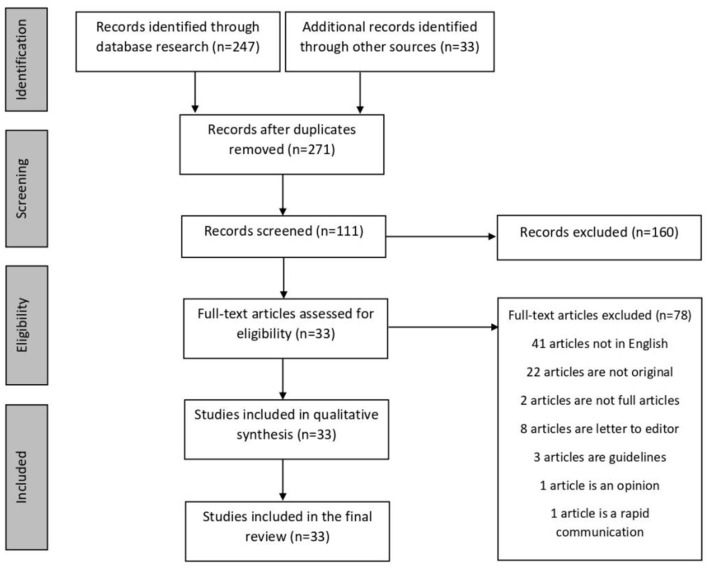
Search algorithm for reviewed articles.

Initially, we identified 280 studies acquired from the database and additional records, of which 169 were excluded because of duplication and a review of the titles and abstract. Additional articles were identified in the reference lists of included studies. We screened the full text of 111 studies for relevance and excluded 78. Finally, 33 papers were selected for final review. Figure 1 shows the PRISMA flow diagram for studies included in the review. A total of 7,559 patients were included. The total number of patients in each study ranged from 5 to 1,590, except for the case reports. The mean age of eight studies ranged from 34.9 to 55.5 years, median age of 20 studies ranged from 32.5 to 73.5 years, and 4 case reports studies ranged from 24 to 74 years. Most of the included studies were from China, with 29 studies (7,528 cases), followed by the United States (US) with 2 (2 cases), Japan with 1 (1 case), and South Korea with 1 (28 cases).

Thirty-one studies (3, 4, 9–37) used reverse transcription-polymerase chain reaction (RT-PCR) as a laboratory-confirmed diagnosis for COVID-19, and one study (38) used RT-PCR and clinical-confirmed diagnosis. Of the remaining study, one study (5) did not mention the diagnosis method. Twenty-five studies (3–5, 9, 11–25, 27–31, 38) reported the specimens for laboratory testing were obtained from a throat swab. Others were from sputum, with four (15–17, 37) from a nasopharyngeal swab and two (4, 37) from a cerebrospinal fluid (CSF) sample (26). Six studies (10, 32–36) did not report the specimen used. Five studies (4, 14–16, 37) used more than one type of specimen collected. Supplementary Table 1 summarizes the characteristics of included studies.

### Neurologic Manifestations in COVID-19

Studies have identified the presence of neurological symptoms in COVID-19 patients. These manifestations were then grouped into several categories based on their symptoms, including nonspecific symptoms, specific symptoms, consciousness disturbance, and skeletal muscle problems. One study (5) particularly examined the neurologic manifestations in COVID-19, with the prevalence of nervous system disease at 36.4% from 214 patients. As for onset, most neurologic symptoms occurred in the early stages of disease (median time, 1–2 days), apart from stroke and impaired consciousness (median time, 8–9 days). The prevalence of neurologic manifestations as reported in these studies are shown in Supplementary Table 1. The overall results of the meta-analysis of neurologic characteristics proportions are shown in Table 1.

**Table 1:** Results of meta-analysis of prevalence based on each neurological manifestation.

**Variables**	**Number of studies**	**Prevalence (%)**	**95% CI (a)**	**Pooled sample size**	***I*^**2**^ (b)**	***p*-value**
Headache	21	10.9	8.62–13.51	6,486	87.8%	<0.0001
Dizziness	6	8.77	5.02–13.43	1,088	81.7%	<0.0001
Nausea with/without Vomiting	13	4.6	3.17–6.27	5,410	82.8%	<0.0001
Cerebrovascular disease	2	4.4	1.92–7.91	435	58.8%	0.1195
Consciousness Disturbance	2	3.8	0.16–12.04	3,848	94.8%	<0.0001
Muscle Problem	25	19.2	15.4–23.2	6,498	92.6%	<0.0001
Cerebrovascular disease comorbidity	13	8.5	4.5–13.5	4148	95.5%	<0.0001

*95% CI: 95% Confidence Interval*.

*I^2^ Index for the degree of heterogeneity*.

### Non-specific Neurologic Manifestations

The primary manifestations of COVID-19 are typically respiratory symptoms. However, physicians have found neurological symptoms at the time of diagnosis as an initial symptom(s). Non-specific symptoms may lead to difficulty of diagnosis when it is the only symptom presented, therefore a differential diagnosis should always be considered to avoid delayed or misdiagnosis.

**Figure 2:**
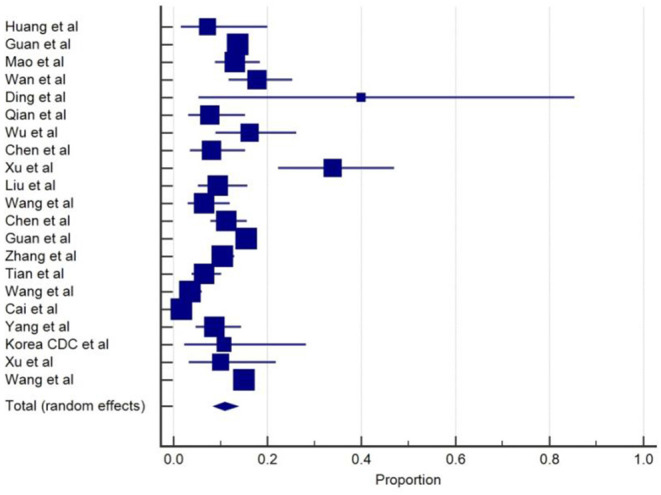
Proportion estimates of headache in COVID-19 patients.

Headache was one of the most common neurologic symptoms in COVID-19 after myalgia, which will be discussed in a later section. Twenty-one studies (3–5, 9, 10, 12–16, 19–21, 23, 27, 28, 30, 33, 35, 37, 38) reported the prevalence of headache ranging from 3.5 to 34% among COVID-19 patients in their baseline characteristics. The overall pooled prevalence of headache was 10.9% (95% CIs: 8.62–13.51) with a high level of heterogeneity (*I*^2^ = 87.8%) from 21 studies with a total number of 6,486 total cases (Table 1). A forest plot of prevalence (%) of headache is included in Figure 2. These findings may be indicative that headache can be found in the early stages of the disease. A retrospective study (37) described that headache was more common among patients with aggravation of illness during follow up (19 vs. 14.6%).

**Figure 3:**
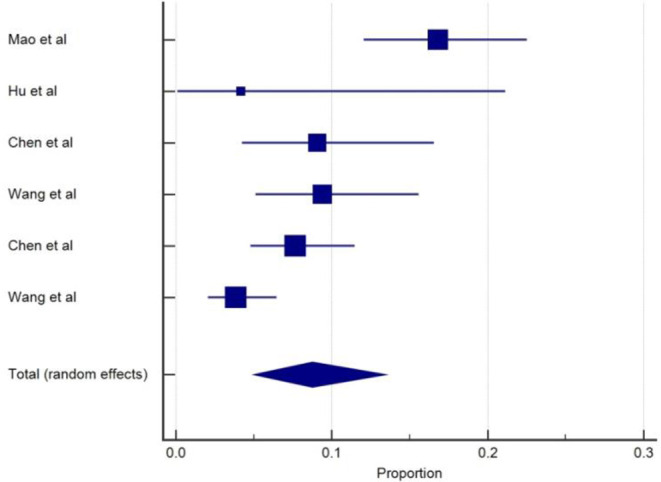
Proportion estimates of dizziness in COVID-19 patients.

Dizziness was reported in 6 studies (5, 11, 13, 16, 19, 27). The overall pooled prevalence of dizziness was 8.77% (95% CIs: 5.02–13.43) with a high level of heterogeneity (*I*_2_ = 81.7%) from six studies, with a total number of 1088 total cases (Table 1). Forest plot of prevalence (%) of dizziness is included in Figure 3. In one study, dizziness (16.8%) was the most common central nervous system manifestation of COVID-19 followed by headache (13.1%). Dizziness and headache were often observed in earlier disease as typical symptoms of COVID-19 (5).

**Figure 4:**
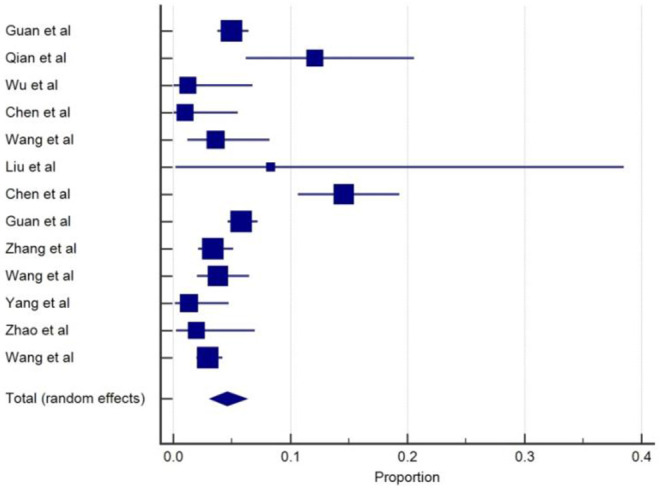
Proportion estimates of nausea with/without vomiting in COVID-19 patients.

Nausea with or without vomiting was reported in 13 studies (4, 12, 13, 16, 17, 19–21, 27, 30, 34, 37, 38) with the prevalence ranging from 1.25 to 8.7%, although vomiting without nausea was reported in one study among non-critically ill patients. The overall pooled prevalence of nausea with or without vomiting was 4.6% (95% CIs: 3.17–6.27), with a high level of heterogeneity (*I*^2^ = 82.8%) from 13 studies with a total number of 5410 total cases (Table 1). The prevalence forest plot (%) of nausea is included in Figure 4.

Most of the studies were conducted during the outbreak period of COVID-19. Therefore, advanced imaging and diagnostic procedures such as magnetic resonance imaging (MRI) and electroencephalography (EEG) were avoided or limited unless the symptoms were specific for a disease (e.g., hemiparesis or seizure). Hence, it is difficult to distinguish the origin of these neurologic symptoms, whether it is caused directly by the virus or indirectly from other organ injury, such as gastrointestinal manifestation (5).

### Specific Neurologic Manifestations

More specific manifestations related to COVID-19 were also observed, such as impairment of smell or taste (hypogeusia) or vision, limb weakness, acute cerebrovascular disease, and seizure. Some specific symptoms were only reported in one study (5) that included impairment of taste (5.6%), smell (5.1%), and vision (1.4%), ataxia (0.5%), and neuralgia (2.3%).

Seizure was less common in COVID-19 and only reported in two case reports (5, 26). However, the diagnosis for seizure was based on clinical founding, without further diagnostic tests. Only one study reported seizure characteristics with a sudden onset of limb twitching, foaming at mouth, and altered consciousness, which lasted for 3 minutes (5). Convulsion in COVID-19 is also associated with an incidence of encephalopathy (26).

In a case report (31) of a 61-year-old female, the patient presented with acute weakness in both legs and severe fatigue progressing within 1 day. Neurological examination showed symmetrical weakness grade 4/5 and areflexia in both legs and feet. The nerve conduction studies showed delayed distal latencies and absent F waves in early course, supporting demyelinating neuropathy, and the patient was diagnosed with Guillain-Barre syndrome (GBS). Interestingly, the onset of weakness precedes the typical COVID-19 symptoms (fever and respiratory symptoms). This report might be an indication that neurologic symptoms could occur in an early stage of the disease.

**Figure 5:**
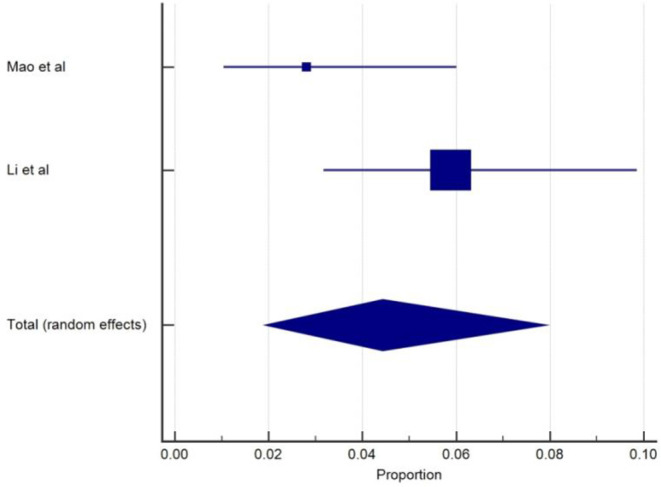
Proportion estimates of cerebrovascular disease in COVID-19 patients.

The incidence of acute cerebrovascular disease (CVD) was reported in two studies (5, 18). The overall pooled prevalence of acute cerebrovascular disease was 4.4% (95% CIs: 1.92–7.91) with a moderate level of heterogeneity (*I*^2^ = 58.8%) from two studies with a total number of 435 total cases (Table 1). A forest plot of prevalence (%) of cerebrovascular disease is included in Figure 5. In a retrospective study (18) among 221 patients with COVID-19, 5.9% of patients had a new onset of CVD during hospitalization stay. Median duration from the first symptoms of infection to a sudden onset of hemiplegia was 9 to 10 days (5, 18). The most common type was ischemic stroke (84.6%), followed by cerebral venous thrombosis (7.7%) and hemorrhage stroke (7.7%). The onset of CVD was more likely to present with those of an older age, severe disease, and history of underlying diseases such as hypertension and diabetes mellitus. This study also showed various inflammatory biomarkers including elevated levels of white blood cells, C-reactive protein, and D-dimers in COVID-19 patients with stroke. In a study by Mao et al. (5), six patients (2.8%) were reported to have CVD and severe patients were more likely to present with CVD than non-severe cases. However, association between COVID-19 and the incidence of cerebrovascular events is lacking and unclear.

### Consciousness Disturbances

**Figure 6:**
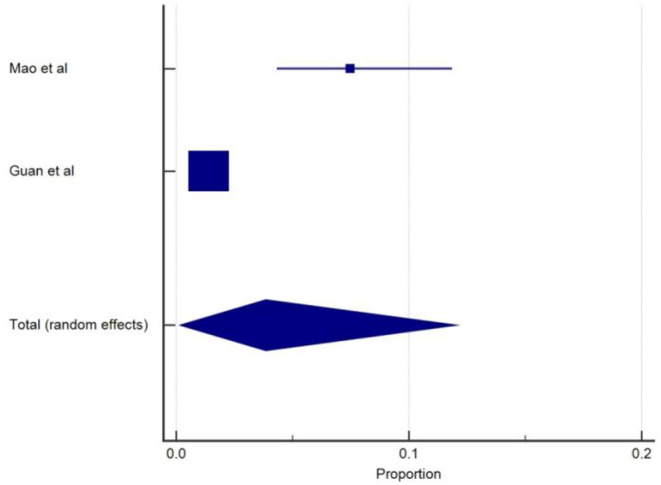
Proportion estimates of consciousness disturbance in COVID-19 patients.

Impaired consciousness was detected in five studies (5, 20, 25, 26, 32). The overall pooled prevalence of consciousness disturbance was 3.8% (95% CIs: 0.16–12.04) with a high level of heterogeneity (*I*^2^ = 94.8%) from two studies, with a total number of 2848 total cases (Table 1). A forest plot of prevalence (%) of consciousness disturbance is included in Figure 6. Three studies were excluded from analysis because those studies were case reports. One study (20) stated that patients with comorbidity on admission were more likely to present with unconsciousness (2.5 vs. 1%). Findings from this limited study have been confirmed in other reports, showing that underlying diseases were associated with the incidence of consciousness disturbance.

A study reported the onset of impaired consciousness (median time, 8 days) to hospital admission was longer compared to other neurologic symptoms (5). In contrast, three case reports (25, 26, 32) described that consciousness disturbance occurs as a presenting symptom of COVID-19. These case reports described altered consciousness on admission was linked to the presence of SARS-CoV-2 infection in the central nervous system, associated with encephalopathy and meningitis/encephalitis. These findings could be helpful to distinguish whether consciousness disturbance has a pure neurologic origin or is caused by an indirect process from organ failure based on the symptom onset, yet the association between consciousness disturbance with SARS-CoV-2 infection remains uncertain.

A case report (32) from the United States reported a 74-year-old man presented to the emergency department with an altered mental status, with prior symptoms of fever, headache, and cough. Electroencephalography shows diffuse slowing and focal slowing sharply contoured waves in the left temporal region, which indicates encephalopathy, and the patient was tested positive for COVID-19. The patient was transferred to ICU with poor outcomes.

An interesting case report (26) from Japan found impaired consciousness followed by convulsion was associated with meningitis/encephalitis, with duration from first symptoms (e.g., headache, fever) to unconsciousness at 9 days. Interestingly, the presence of SARS-CoV-2 infection in this case was detected only through CSF specimen and negative from throat swab. Loss of consciousness associated with seizure was also reported in another retrospective study (5, 26).

Altered mental status was also related to a rare complication of viral infection. This report by Poyiadji et al. (25) described a 50-year-old female who was brought in with 3 days history of fever, cough, and altered mental status. The laboratory-confirmed positive for COVID-19. CSF findings were normal. Non-contrast head CT found hypoattenuation within the bilateral medial thalamic, whereas an MRI showed rim enhancing lesions with hemorrhage within the bilateral thalami, medial temporal lobes, and sub-insular regions. This is the first reported case of COVID-19–associated acute necrotizing encephalopathy (ANE).

### Skeletal Muscle Problems and Indicator of Muscle Injury in COVID-19

**Figure 7:**
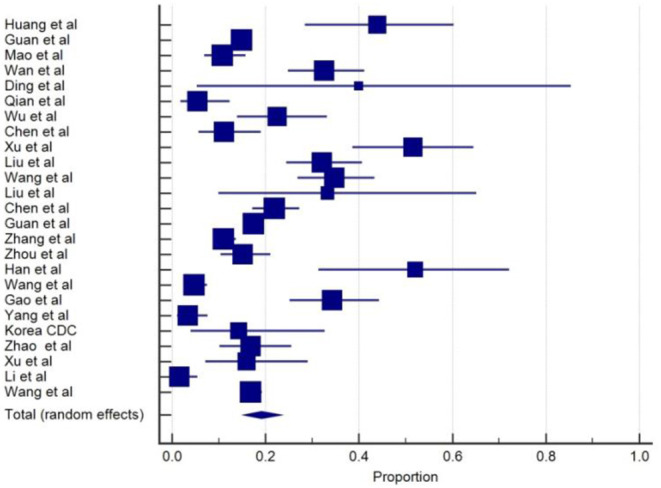
Proportion estimates of muscle problem in COVID-19 patients.

SARS-CoV-2 infection appears to affect the muscles and cause skeletal muscle problems. Muscle injury and myalgia as a manifestation of COVID-19 were reported in 25 from the total of 33 studies and commonly appears alongside several other symptoms with the prevalent, ranging from 2 to 52% (3–5, 9, 10, 12–17, 19–22, 24, 27, 29, 30, 33–38). The overall pooled prevalence of skeletal muscle problems was 19.2% (95% CIs: 15.4–23.2) with a high level of heterogeneity (*I*^2^ =92.6%) from 25 studies with a total number of 6,498 total cases (Table 1). A forest plot of prevalence (%) of skeletal muscle problems of studies is included in Figure 7.

Some COVID-19 patients showed malaise, muscle soreness, and elevated muscle enzyme levels, which may be related to the inflammation and muscle injury caused by the virus. The higher levels of creatine kinase (CK) levels in blood have been generally considered to be an indicator of muscle damage and inflammatory response (39). From the total, 25 studies that reported muscle injury as a manifestation of COVID-19. Of these, 16 studies had patients with COVID-19 who also had higher levels of CK serum. However, some of these studies used different standards in determining elevated levels of CK serum. Several studies were using range >185 units per liter (U/L) for elevated CK levels (3, 13, 14, 17, 22), four studies set the value at >200 U/L (4, 9, 24, 30), and one study set the value at >310U/L (12). Of these, six studies (5, 16, 19, 21, 27, 29) mentioned an elevation in CK levels but did not report the normal value to assess elevated CK levels. Seven studies (3–5, 9, 16, 19, 27) comparing CK levels between severe and non-severe cases found that CK levels tend to be higher in severe cases, including ICU patients, or among deceased patients.

Of the included studies, the authors did not perform electromyography (EMG) or other diagnostic tests to indicate myopathic changes. Therefore, it remains difficult to differentiate between inflammation-related muscle injury and other neuromuscular disorders or myopathy.

### Neurological Features in Severe COVID-19

It has been investigated whether, for those in the severe stage of the disease, clinical deterioration may be associated with neurologic events. A total of six6 studies (4, 5, 9, 23, 28) reported neurologic symptoms were more common in severe cases. Mao et al. (5) reported that patients with more severe disease were more likely to present with nervous system symptoms (45.5 vs. 30.2%, *p* < 0.05); including impaired consciousness (14.8 vs. 2.4%), acute cerebrovascular events (5.7 vs. 0.8%), and muscle injury (19.3 vs. 4.8%). One population-based survey (4) of laboratory-confirmed COVID-19 patients reported nationwide clinical characteristics of COVID-19 in 1,099 patients. In severe cases, patients were more likely to present with headache (15 vs. 13.4%), nausea or vomiting (6.9 vs. 4.6%), myalgia or arthralgia (17.3 vs. 14.5%), and CVD comorbidity (2.3 vs. 1.2%). Following SARS-CoV-2 infection, patients with neurological involvement were more likely to require intensive care unit (ICU) interventions (5, 16).

A study categorized neurologic findings based on their system, including central and peripheral nervous systems. In the severe group, the central nervous system symptoms (e.g., dizziness, headache, cerebrovascular events) were more common compared to peripheral nervous system manifestations (30.7 vs. 8%).

**Figure 8:**
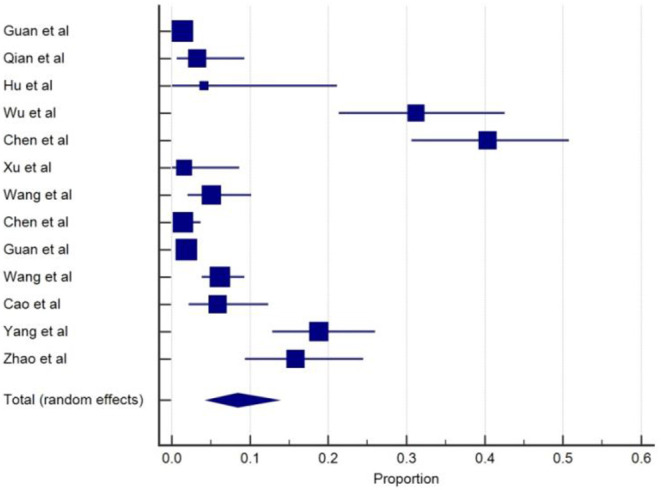
Proportion estimates of cerebrovascular disease comorbidity in COVID-19 patients.

Patients with severe disease were also more likely to experience myalgia compared to the non-severe group (17.3% vs. 14.5%) (4). Similarly, six studies (3, 5, 9, 27, 29, 37) also reported that muscle problem was more common among severe cases or non-survivors. In a retrospective study (20), COVID-19 patients with comorbidity were more likely to have muscle pain. In terms of comorbidity, cerebrovascular disease (CVD) was commonly reported as a neurologic comorbidity in COVID-19 patients. We found 13 studies (4, 11–14, 16, 19, 20, 27, 29, 30, 34, 38) that reported the presence of CVD as an underlying disease in COVID-19 patients. The rate of CVD comorbidity ranged from 1.4 to 40 %. The overall pooled prevalence of CVD comorbidity was 8.5% (95% CIs: 4.5–13.5) with a high level of heterogeneity (*I*^2^ = 95.5%) from 13 studies with a total number of 4,148 cases (Table 1). A forest plot of prevalence (%) of CVD comorbidity of studies is included in Figure 8.

CVD comorbidity was also a predictive factor of poor outcomes. In a retrospective study (27) on 339 hospitalized COVID-19 patients, the patients' prognostic factors were evaluated based on a 4-week follow-up; 21 (6.2%) patients had CVD comorbidity and this was more prevalent in patients who died (10/65 or 15.6%). Similarly, two studies (19, 29) reported that patients with CVD comorbidity on admission had a higher mortality rate (4 vs. 0%) and was more prevalent among non-survivors (17.6vs. 3.5%), respectively. A complication of hypoxic encephalopathy occurrence following COVID-19 was also observed in 20% of patients and was likely to occur in patients who died (20% vs. 1%) than patients who survived (19).

## Discussion

As a newly emerging disease, COVID-19 has become a pandemic disease since its outbreak in December 2019. The infection caused by SARS-CoV-2 mainly targets the respiratory tract. However, studies also reported the involvement of the nervous system as presenting symptoms, especially in different stages of disease (40). During the early phase of the disease, symptoms were commonly mild or asymptomatic. Therefore, the diagnosis of the disease was often difficult at the time of presentation (4). Patients may be referred to the neurologic clinic without respiratory symptoms, with infection that may be hard to detect or that could be misdiagnosed.

### Pathophysiology of Neurologic Manifestations in COVID-19

SARS-CoV-2 used the angiotensin-converting enzyme 2 (ACE2) receptor to enter the host cells, the same as SARS-CoV infection in 2003. The pathologic mechanism COVID-19 has on the nervous system can happen through several pathways, including the hematogenous pathway, retrograde neural pathway, hypoxia, immune injury, and ACE-2 enzyme (41). The virus entering blood circulation can cause the immune system to produce cytokine as a physiological response; the increasing cytokine production may cause an increase in blood-brain barrier permeability, thereby facilitating the virus to enter the CNS (41). This may explain why patients with more severe COVID-19 might have cytokine storm syndrome.

A case report of a rare complication of viral infections has been related to intracranial cytokine storms, which result in blood-brain barrier disruption without direct viral invasion. This theory has been linked to the first reported case of COVID-19-associated acute necrotizing encephalopathy (ANE) (42).

A recent study on COVID-19 patients with a neurologic manifestation found that some patients had smell impairment (5). This finding presents the fact that COVID-19 can invade the central nervous system through the olfactory nerve and gain access to the central nervous system (41). Hypoxia injury happens when a virus begins to proliferate in the lung cells, causing alveolar gas exchange disorder and thus leading to hypoxia of the CNS and increasing the rate of anaerobic metabolism, which leads to the accumulation of acid and can cause cerebral vasodilation, interstitial edema, swelling of brain cells, or obstruction of cerebral blood flow that leads to headache as a result of congestion and ischemia (5)^.^

The past study suggested the neurotropic potential of COVID-19 (43). The neurotropic virus had the ability to incite the activation of glial cells and invoke a proinflammatory state. Furthermore, an elevated level of proinflammatory cytokine in serum may cause chronic inflammation and lead to damage in skeletal muscle and brain (5, 40). ACE-2 is a protecting factor that plays a major role in anti-atherosclerosis and blood pressure regulation. COVID-19's capability to bind ACE-2 receptors may result in elevated blood pressure and increase the feasibility of cerebral hemorrhage (40).

### Laboratory Abnormality and Neurologic Manifestations in COVID-19

Severe SARS-CoV-2 infection has been associated with increased immune-inflammatory response, including higher white blood cell counts, neutrophil counts, lower lymphocyte counts, and increased C-reactive protein levels compared with non-severe infection. Some laboratory findings were also associated with the neurologic manifestations of the disease. Patients with central nervous symptoms involvement had lower lymphocyte levels, platelet counts, and higher blood urea nitrogen levels compared with those without CNS symptoms. However, there were no significant differences in laboratory findings of patients with PNS manifestations and those without PNS (5). This finding may be a link to the immunosuppression among patients with CNS symptoms, especially in severe patients.

Skeletal muscle injury was described as skeletal muscle pain or myalgia with an elevated level of serum creatine kinase (CK) more than 200 U/L as a manifestation of an increased inflammatory response (5). The cellular disturbances caused by the infection or direct muscle injury by the virus can induce creatinine kinase to leak from intra cells into the blood. Assessment of serum CK levels are a valuable indicator of the occurrence of muscle and tissue damage due to disease or trauma. This finding may be associated with the ACE-2 receptor in skeletal muscle (5, 40).

Additionally, the coagulation system was also affected by the SARS-CoV-2 infection, causing the elevated level of D-dimer more than ≥0.5 mg/L and platelet abnormalities, which increases the risk of cerebrovascular events among patients. Elderly populations are at high risk of account for the majority of strokes, especially in more severe patients. Moreover, some studies reported the increased level of D-dimer was more prevalent in more severe COVID-19, which could be the source of embolic cerebrovascular diseases (4, 18, 40).

### Neurologic Symptoms Characteristics in COVID-19

One population-based survey of laboratory-confirmed COVID-19 patients reported nationwide clinical characteristics of the disease in 1,099 patients. The study found some of the common symptoms in COVID-19 patients, including fever, cough, nausea, fatigue, myalgia, and headache (4). Patients with fever or headache may present to the neurology clinic after initially being ruled out of COVID-19 by routine examination. However, several days later, patients presented typical COVID-19 symptoms such as cough, throat pain, lower lymphocyte count, and pneumonia appearance on lung imaging. These findings showed that COVID-19 often presents with non-specific symptoms and leads to delayed and inappropriate management (40).

Mao et al. (5) reported that neurologic symptoms in COVID-19 can range from specific symptoms (e.g., hypogeusia, hyposmia, or stroke) to more nonspecific symptoms (e.g., headache, impaired consciousness, dizziness, or myalgia). Nonspecific symptoms were more commonly present in mild or early stages of the disease. However, future studies are required to identify which manifestations are truly neurologic in origin or just a response of systemic inflammation of the disease in patients (44).

As known from previous studies, COVID-19 puts the elderly population and patients with pre-existing comorbidity and prior neurological conditions (e.g., history of cerebrovascular disease) at a higher risk of developing more severe symptoms, such as encephalopathy, altered mental status, and new onset of stroke on admission. A recent study reported four COVID-19 patients with acute stroke as a presenting symptom. The patients admitted to the hospital with a positive PCR test and imaging confirmed acute stroke. The patients presented neurological symptoms such as altered mental status, facial drop, slurred speech, hemiparesis, hemiplegic, and aphasia. The pathophysiology behind this may be related to the infection or hypoxia that leads to brain ischemia (45). Severe COVID-19 patients were also more likely to develop more specific neurologic manifestations. Moreover, underlying cerebrovascular disease is related to poor prognosis (5, 18).

COVID-19 may also invade and disrupt the intracranial component. Researchers have detected the incidence of encephalopathy and meningitis in COVID-19 with symptoms of impaired consciousness and seizures (22, 25, 26). This may happen because of COVID-19 binding to ACE-2 receptors in the brain and causing damage in the brain tissue, thus leading to impaired consciousness and seizures (26). COVID-19 also induces the intracranial cytokine storms, which result in the breakdown of the blood-brain barrier and leads to damage in brain tissue (25). Importantly, the virus can be found in the cerebrospinal fluid through nucleic acid examination using PCR. Some studies even reported the central nervous system manifestations preceded respiratory symptoms (25). Therefore, physicians should stay aware and look at the potential signs of intracranial or other organ involvement.

### Neurologic Symptoms Characteristics in SARS-CoV and MERS-CoV

Similar to SARS-CoV-2 that caused COVID-19, Severe Acute Respiratory Syndrome Coronavirus (SARS-CoV) patients also present several neurological symptoms. According to previous studies, myalgia (45–61%), headache (20–56%), dizziness (4.2–43%), nausea, and vomiting (20–35%) are among the neurological symptoms found in SARS-CoV patients, which are similar to neurological symptoms frequently found in COVID-19 patients (46). Several studies also reported neurological manifestations such as stroke, myopathy, polyneuropathy, and rhabdomyolysis in SARS-CoV patients (47–49). Middle East Respiratory Syndrome Corona Virus (MERS-CoV) patients present several neurological symptoms similar to SARS-CoV and COVID-19, such as myalgia, headache, nausea, and vomiting (50). The neurological complication of MERS-CoV reported in previous studies consists of stroke, Bickerstaff's encephalitis (BBE), Guillain-Barré syndrome (GBS), and polyneuropathy (51–53).

### Limitations

Our study has several limitations. First, the search keyword used in this systematic review was limited to the terms of “characteristics,” so there is a possibility that relevant studies were missed by the search. Second, our study was also limited to the English language. Third, there were variations among laboratory value findings in the studies included. Some studies used a different standard of laboratory normal value range, which may lead to misinterpretation. Fourth, several studies included in this research are lacking in severity degree of neurological symptoms. Another important limitation was the variation in the methodologic quality of the included studies. Most of the included studies were observational studies and the data used was obtained from medical records. Therefore, comparability among literature was also limited. Furthermore, the majority of the studies retrieved were from China, which led to a lack of data from other countries. Therefore, further studies are needed to provide a different perspective from neurologic findings of patients with COVID-19 from other countries.

## Conclusion

As a pandemic, COVID-19 has become common globally. Physicians are expected to be prepared for and confronted with these patients in the upcoming period. The disease can range from mild disease with asymptomatic or non-specific symptoms to severe disease with respiratory distress. The neurologic symptoms are more commonly found in the later or severe stage of the disease and may not be found at the early stage or mild disease, yet physicians should stay vigilant and aware of those symptoms as signs of nervous systems complicity (40).

It is hoped that this brief review may provide a spectrum of neurologic manifestations in COVID-19. Therefore, early diagnosis and management can prevent further deterioration from neurologic complications in the later stage of disease. Our review could be a reference for physicians in management and detection of neurological symptoms in COVID-19 patients.

## Author Contributions

RP: study concept and design, supervision, writing of the initial draft, and final revision. VW: study concept and design, writing of the initial draft, data extraction, analysis, and interpretation. RB and PN: full text review, analysis and interpretation, and manuscript preparation. AA: abstract screening, data extraction, analysis, and interpretation.

## Conflict of Interest

The authors declare that the research was conducted in the absence of any commercial or financial relationships that could be construed as a potential conflict of interest.
