# Supplementary material for: Neurologic Characteristics in Coronavirus Disease 2019 (COVID-19): A Systematic Review and Meta-Analysis
Source: Front Neurol. 2020 May 29;11:565. doi: 10.3389/fneur.2020.00565 (PMC7273516; doi:10.3389/fneur.2020.00565)
Supplement: Supplementary file 1 [file Table_1.docx]

|  | **Table 1.** Studies Included in Systematic Review | | | | | | | | |
| --- | --- | --- | --- | --- | --- | --- | --- | --- | --- |
| Authors | | Study Type | Country Location | Study Group | Age, Median (IQR, y) or Mean ± SD | Neurologic Symptoms No. (% of total) | Key Findings | Limitations | Study Quality Level |
| Huang et al,^3^ 2020 | | Prospective Cohort | China | 41 patients with RT-PCR positive COVID-19 from throat swab | 49 (41- 58 y) | - Headache, 3 (8%) - Myalgia, 18 (44%) | - Subjects were 13/41 (32%) ICU patients and 28/41 (68%) non-ICU patients - Headache was more common in non-ICU patients (12% vs 0%) - Myalgia was more common in ICU patients (54% vs 39%) - 13/40 (33%) patients had elevated CK levels (> 185 U/L) and more common in ICU patients (46% vs 26%) | - Not specifically studied neurological symptoms - No clear definition of symptoms - Diagnostic test was limited | 2 |
| Guan et al,^4^ 2020 | | Cross Sectional | China | 1099 patients with RT-PCR positive COVID-19 from nasopharyngeal and throat swab | 47 (35-58 y) | - Headache, 150 (13.6%) - Nausea or Vomiting, 55 (5 %) - Myalgia or Arthralgia, 164 (14.90%) | - Data obtained from 522 hospitals in 30 provinces in mainland China - Based from disease severity, 926/1099 (84%) were mild and 173/1099 (16%) were severe cases - In severe cases, patients were more likely to present with headache (15% vs 13.4%), nausea or vomiting (6.9% vs 4.6%), myalgia or arthralgia (17.3% vs 14.5%), and CVD comorbidity (2.3% vs 1.2%) - 15/1099 (1.40%) patients had CVD comorbidity - 90/657 (13.7%) patients had elevated CK levels (>200 U/L) and more common in severe cases (19% vs 12.5%) - 260/560 (46.4%) patients had elevated D-dimer ( ≥ 0.5 mg/L) and more common in severe cases (59.6% vs 43.2%) | - No clear definition of symptoms - Not specifically studied neurological manifestation - Diagnostic test was limited | 4 |
| Mao et al,^5^ 2020 | | Retrospective Cohort | Wuhan, China | 214 patients with RT-PCR positive COVID-19 from throat swab | Mean Age (y) ± SD: 52.7y ± 15.5 | Central Nervous Symptoms:   - Dizziness, 36 (16,8%) - Headache, 28 (13.1%) - Impaired consciousness, 16 (7.5%) - CVD, 6 (2.8%) - Ataxia, 1 (0.5%) - Epilepsy, 1 (0.5%)   Peripheral Nervous Symptoms:   - Hypogeusia, 12 (5.6%) - Hyposmia, 11 (5.1%) - Hypopsia, 3 (1.4%) - Neuralgia, 5 (2.3%) - Muscle injury, 23 (10.7%) | - Based from disease severity, 126/214 (58.9%) were mild and and 88/214 (41.1%) were severe cases - Patients with severe COVID-19 were more likely to present with nervous system symptoms (45.5% vs 30.2%, p<0.05), impaired consciousness (14.8% vs 2.4%, p<0.001), CVD (5.7% vs 0.8%, p<0.05), and muscle injury (19.3% vs 4.8%, p<0.001) - 78/214 (36.4%) patients had nervous system symptoms classified into: CNS, 53(24.8%); PNS, 19 (8.9%); and skeletal muscular symptoms, 23(10.7%). - CK levels was higher in severe patients (83 vs 59, p=0.004) - Most neurologic symptoms occurred in the early stages of disease (median time, 1-2 days), Apart from CVD and impaired consciousness (median time, 8-9 days) - 40/214 (18.7%) patients were admitted to ICU for severe neurological manifestations. | - No clear definition of symptoms - Incomplete data or observation as the study had ended - Diagnostic test was limited | 3 |
| Wan et al,^9^ 2020 | | Retrospective Cohort | Chongqing, China | 135 hospitalized patients with RT-PCR positive COVID-19 from throat swab | 47 (36-55 y) | - Headache, 24 (17.7%) - Myalgia, 44 (32.5%) | - Based from disease severity, 95/135 (70.4%) were mild and 40/135 (29.6%) were severe cases - In severe cases, headache (27.5% vs 24.2%, p>0.05) and myalgia (47.5% vs 26.3%, p<0.05) were more common. - 10/135 (7.4%) patients had elevated CK levels (>200 U/L) and more common in severe cases (17.5% vs 3%) | - No clear definition of symptoms - Not specifically studied neurological manifestation - Diagnostic test was limited | 3 |
| Ding et al,^10^ 2020 | | Retrospective Case series | Wuhan, China | 5 COVID-19 patients. Diagnosis method was not reported | (39-66 y) | - Headache, 2 (20%) - Myalgia, 2 (20%) | - 5/5 (100%) all subjects had co-infected with influenza A (60%) and B (40%) virus | - Small sample size - No clear definition of symptoms - Not specifically studied neurological manifestation - Diagnostic test was limited | 4 |
| Qian et al,^38^ 2020 | | Retrospective Cohort | Zhejiang, China | 88 patients with RT-PCR positive from throat swab and 3 cases of clinical-confirmed COVID-19 | 50 (36.5-57 y) | - Headache, 7 (7.69%) - Nausea, 11 (12.09%) - Vomiting, 6 (6.59%) - Myalgia, 5 (5.49%) - Back discomfort, 3(3.3%) | - Based from disease severity, 82/91 (90%) were mild and and 9/91 (10%) were severe cases - 3/91 (3.30%) patients had CVD comorbidities | - Small sample size - No clear definition of symptoms - Not specifically studied neurological manifestation - Diagnostic test was limited | 3 |
| Hu et al,^11^ 2020 | | Retrospective case series | Nanjing, China | 24 patients with RT-PCR positive COVID-19 from throat swab | 32.5 (5-95 y) | - Dizziness, 1 (4.2%) - Arthralgia, 1 (4.2%) | - 1/24 (4.2%) patients had CVD comorbidity | - No clear definition of symptoms - Not specifically studied neurological manifestation - Diagnostic test was limited | 4 |
| Wu et al,^12^ 2020 | | Cross sectional | Jiangsu, China | 80 patients with RT-PCR positive COVID-19 from throat swab | 46.1 (30.7–61. 5 y) | - Headache, 13 (16.25%) - Nausea, 1 (1.25%) - Vomiting, 1 (1.25%) - Myalgia, 18 (22.50%) | - 25/80 (31.25%) patients had CVD comorbidity - 1/80 (1.25%) patients had nervous system disease comorbidity - 18/80 (22.5%) patients had CK levels (>310 U/L) above normal range | - No clear definition of symptoms - Not specifically studied neurological manifestation - Diagnostic test was limited | 4 |
| Chen N,^13^ et al 2020 | | Cross Sectional | China | 99 patients with RT-PCR positive COVID-19 from throat swab | Mean Age (y) ± SD: 55.5 ± 13.1 | - Dizziness, 9 (9%) - Headache, 8 (8%) - Nausea and Vomiting, 1 (1%) - Myalgia, 11 (11%) | - 40/99 (40%) patients had CVD comorbidities - 1/99 (1%) patients had nervous system disease - 13/99 (13%) patients had elevated CK levels (> 185 U/L) | - No clear definition of symptoms - Not specifically studied neurological manifestation - Diagnostic test was limited | 4 |
| Xu et al,^14^ 2020 | | Retrospective case series | China | 62 patients with RT-PCR positive COVID-19 from throat swab and sputum | 41 (32-52 y) | - Headache, 21 (34%) - Myalgia, 32 (52%) | - 1/62 (2%) patients had CVD comorbidity - 5/62 (8%) patients had elevated CK levels (> 185 U/L) | - No clear definition of symptoms - Not specifically studied neurological symptoms - Diagnostic test was limited | 4 |
| Liu et al,^15^ 2020 | | Cross sectional | China | 137 patients with RT-PCR positive COVID-19 from throat swab and sputum | 57 (20-83y) | - Headache, 13 (9.5%) - Myalgia, 44 (44%) | - 27/137 (19.7%) patients had underlying disease | - No clear definition of symptoms - Not specifically studied neurological symptoms. - Diagnostic test was limited | 4 |
| Wang et al,^16^ 2020 | | Retrospective Cohort | China | 138 patients with RT-PCR positive COVID-19 from throat swab and sputum | 56 (22-92 y) | - Dizziness, 13 (9.4%) - Headache, 9 (6.5%) - Vomiting, 5 (3.6%) - Myalgia, 48 (34.8%) | - Subjects were 36/138 (26%) ICU patients and 102/138 (74%) non-ICU patients - Patients admitted to ICU were more likely to present with dizziness (22.2% vs 4.9%, p<0.05) and comorbidity of CVD (16.7% vs 1%, p=0.001) - 7/138 (5.1 %) patients had CVD comorbidity - CK levels was higher in ICU patients (102 vs 87, p=0.08) | - No clear definition of symptoms - Not specifically studied neurological manifestation - Diagnostic test was limited | 3 |
| Liu et al,^17^ 2020 | | Retrospective Case series | China | 12 patients with RT-PCR positive COVID-19 from throat swab | 62.5  (10-72 y) | - Nausea and Vomiting, 1 (8.3%) - Myalgia, 4 (33.3%) | - 1/6 (16.6%) patients had elevated CK levels (> 185 U/L). | - Not specifically studied neurological symptoms - No clear definition of symptoms - Diagnostic test was limited - Insufficient data may result to a biased understanding of the disease. | 4 |
| Li et al,^18^ 2020 | | RetrospectiveCohort | Wuhan, China | 221 patients with RT-PCR positive COVID-19 from throat swab | 73.5 (57-91 y) | - Cerebrovascular disease, 13/221 (5.9%), - Ischemic Stroke, 11/13 (84.6%) - CVST, 1/13 (7.7%) - Cerebral Hemorrhage, 1/13 (7.7%) | - Median durations from first symptoms of infection to CVD onset were 10 days (IQR 1-29) - The onset of CVD was more likely to present with older age (71.6y vs 52.1y, p<0.05), severe COVID-19 (84.6% vs 39.9%, p<0.01), and underlying diseases such as hypertension (p<0.001) or diabetes mellitus (p<0.01) - Patients with CVD had more increased in some laboratory findings, including higher white blood cell (p<0.001), and C-reactive protein levels (p<0.01), and D-dimer levels (p<0.001) | - Not specifically studied neurological symptoms - No clear definition of symptoms - Diagnostic test was limited | 3 |
| Chen et al,^19^ 2020 | | Cross sectional | Wuhan, China | 274 patients with RT-PCR positive COVID-19 from throat swab | 62.0 (44.0-70.0 y) | - Dizziness, 21 (7,6%) - Headache, 31 (11,3 %) - Myalgia, 60 (21,8%), - Nausea, 24 (8,7%) - Vomiting, 16 (5,8%) | - 113/274 (41%) died and 161/274 (59%) survives during hospitalization stay. - 4/274 (1%) patients had CVD comorbidity and was more common among deceased patients (4% vs 0%) - 24/274 (9%) patients had complication of hypoxic encephalopathy and was more common among deceased patients (20% vs 1%) - CK levels was higher in deceased patients (189 vs 84) | - Not specifically studied neurological symptoms - No clear definition of symptoms - Diagnostic test was limited - There are missing data or important tests that might lead to bias of clinical characteristics | 4 |
| Guan et al,^20^ 2020 | | Retrospective Cohort | China | 1590 patients with RT-PCR positive COVID-19 from throat swab | Mean Age (y) ± SD: 48.9±16.3 | - Headache, 205/1328 (15.4 %) - Unconsciousness 20/1421 (1.4%) - Nausea/vomiting 80/1371 (5.8%) - Myalgia/arthralgia 234/1338 (17.5%) | - 399/1590 (25.1%) had at least one comorbidity. - Patients with comorbidity were more likely to present with headache (16.6% vs 15.1%), unconsciousness (2.5% vs 1%), nausea/vomiting (10.4% vs 4.3%) and myalgia/arthralgia (18.1% vs 17.3%) - 30/1590 (1.9%) patients had CVD comorbidity. | - Not specifically studied neurological symptoms - No clear definition of symptoms - Diagnostic test was limited | 3 |
| Zhang et al,^21^ 2020 | | Retrospective Cohort | Zhejiang, China | 645 patients with RT-PCR positive COVID-19 from throat swab | Mean Age (y) ± SD:  Normal imaging findings= 34.90±14.20; Abnormal imaging findings = 46.65±13.82 | - Headache, 67 (10.3%) - Nausea and vomiting, 22 (3.40%) - Myalgia, 71 (15.2%) | - 72/645 (11%) patients had normal imaging, while 573/645 (89%) had abnormal imaging findings - Headache were more common in those abnormal imaging findings (11.3% vs 2.8%) - CK levels were higher in those with abnormal imaging findings (73.0 vs 62.5, p<0.05) | - Not specifically studied neurological symptoms - No clear definition of symptoms - Diagnostic test was limited | 3 |
| Zhou et al,^22^ 2020 | | Retrospective Cohort | Wuhan, China | 191 patients with RT-PCR positive COVID-19 from throat swab | 56 (46.0-67.0 y) | - Myalgia, 29 (15%) | - 22/168 (13%) had elevated CK levels (>185 U/L) - CK levels were more common (21% vs 9%, p<0.05) and higher (73.0 vs 62.5, p<0.05) among non-survivor patients | - Not specifically studied neurological symptoms - No clear definition of symptoms - Diagnostic test was limited | 3 |
| Tian et al,^23^ 2020 | | Retrospective Cohort | Beijing, China | 262 patients with RT-PCR positive COVID-19 from throat swab | 47.5(1–94 y) | - Headache, 17 (6.5%) | - Based from disease severity, 216/262 (82.4%) were common and and 46/262 (17.6%) were severe group - Headache was present in 3/46 (6.5%) patients with severe group and 14/216 (6.5%) patients with common group | - No clear definition of symptoms - Not specifically studied neurological symptoms - Diagnostic test was limited | 3 |
| Han et al,^24^ 2020 | | Retrospective Cohort | Shaanxi, China | 25 adult patients with RT-PCR positive COVID-19 from throat swab | 44(22-70 y) | - Myalgia, 13 (52%) | - In adults, myalgia or fatigue were observed more frequently with no significant difference (52% vs 0%, P=0.25). - 1/25 (4%) adult patients had elevated CK levels (>200 U/L) | - Small sample size - No clear definition of symptoms - Not specifically studied neurological symptoms - Diagnostic test was limited | 3 |
| Poyiadji et al,^25^ 2020 | | Case  Report | Detroit, United States | A female airline worker patients with RT-PCR positive COVID-19 from throat swab | 50 year old | - 3 days history of fever, cough, and altered mental status | - This is the first reported case of COVID-19–associated acute necrotizing hemorrhagic encephalopathy - Imaging findings: Noncontrast head CT images, symmetric hypoattenuation within the bilateral medial thalamic; Images from brain MRI, hemorrhagic rim enhancing lesions within the bilateral thalami, medial temporal lobes, and subinsular regions - CSF finding were normal and negative for bacterial culture, HSV-1 and 2, Varicella zoster, and West Nile virus | - Diagnostic test was limited | 5 |
| Moriguchi et al,^26^ 2020 | | Case Report | Japan | A man with RT-PCR positive COVID-19 from spinal fluid, and negative from nasopharyngeal swab | 24 year old | - Headache, fever, fatigue (day 1) - Unconsciousness followed by seizure (day 9) | - This is the first reported case of COVID-19 associated meningitis/encephalitis - Durations from first symptoms of infection to unconsciousness were 9 days - Clinical findings were GCS of 6 and neck stiffness - Imaging findings: chest CT, ground glass opacity on pulmonary lobes indicates pneumonia; brain MRI, right lateral ventriculitis and encephalitis mainly on right mesial lobe and hippocampus - Cerebrospinal fluid was clear, colorless, initial pressure > 320 mmH2O, cell count was 12/μL (10 mononuclear and 2 polymorphonuclear cells without red blood cells ) | - Patient outcome was not reported as the study was still ongoing | 5 |
| Wang et al,^27^ 2020 | | Retrospective Cohort | China | 339 patients with RT-PCR positive COVID-19 from throat swab | 65 (65-76 y) | - Dizziness, 13 (3.8%) - Headache, 12 (3.5%) - Myalgia, 16 (4.7%) - Nausea, 13 (3.8%) | - 21/339 (6.2%) patients had CVD comorbidity and more prevalent in deceased patients 10/65 (15.6%) - In survivors, patients were more likely to present with dizziness (4% vs 3.1%), headache (3.5% vs 0%), myalgia (5.5% vs 1.6%), and nausea (4.4% vs 1.6%) - CVD comorbidity was predictive factor of poor outcomes (HR 3.26, CI 1.66-6.40, P = 0.001) - CK levels were higher among deceased patients (84 vs 60 , p=0.005) | - Not specifically studied neurological symptoms - No clear definition of symptoms - Diagnostic test was limited | 3 |
| Cai et al,^28^ 2020 | | Retrospective Cohort | China | 298 patients with RT-PCR positive COVID-19 from throat swab | 47 (33-61y) | - Headache, 5 (2.08%) | - Based from disease severity, 240/298 (80.5%) were common and and 58/298 (19.5%) were severe group - Headache was more common among non-severe group compared to severe group (5/240 [12%] vs 0/58 [0%], p >0.05) - Compared to non-severe patients, CK levels significantly increased among severe patients (64.5 vs 87, p=0.006). | - Not specifically studied neurological symptoms - No clear definition of symptoms - Diagnostic test was limited | 3 |
| Cao et al,^29^ 2020 | | Prospective Cohort | China | 102 patients with RT-PCR positive COVID-19 from throat swab | 54 (37-67 y) | - Myalgia, 35 (34.4%) | - Myalgia was more common among non-survivors group (34.6% vs 29.4%, p=0.641) - 6/102 (5.9 %) patients had CVD comorbidity and more common among non-survivors group 3/17 17.6 % vs 3.5%, p=0.090) | - Not specifically studied neurological symptoms - No clear definition of symptoms - Diagnostic test was limited | 2 |
| Yang et al,^30^ 2020 | | Retrospective Cohort | Wenzhou, China | 149 patients with RT-PCR positive COVID-19 from throat swab | Mean Age (y) ± SD:  45.11 ± 13.35 | - Headache, 13 (8.72%) - Nausea and Vomiting, 2 (1.34%) - Myalgia, 5 (3.36%) | - 28/149 (18.79%) have cerebrovascular underlying disease - 12/149 (8.05%) patients had elevated CK levels (>200 U/L) | - Not specifically studied neurological symptoms - No clear definition of symptoms - Diagnostic test was limited - Incomplete data or observation as the study had ended | 3 |
| Zhao et al,^31^ 2020 | | Case Report | Shanghai, China | A female with RT-PCR positive COVID-19 from throat swab | 61 year old | - Patient presented with acute weakness in both legs and severe fatigue progressing within 1 day | - Patient had travel history returned from Wuhan, China 4 days earlier before the weakness onset - Neurological examination showed symmetrical weakness grade 4/5 and areflexia in both legs and feet - Nerve conduction studies (day 5) showed delayed distal latencies and absent F waves in early course, supporting demyelinating neuropathy and was diagnosed with GBS - Fever and respiratory symptoms occurred after GBS symptoms (day 8) - All symptoms resolved and patient discharged at day 30 | - Diagnostic test was limited | 5 |
| Filatov et al,^32^ 2020 | | Case Report | United States of America | A man with COVID-19 with RT-PCR positive COVID-19 | 74 year old | - The patient presented with fever and cough, and then was discharged with suspicion of the COPD exacerbation. - Within 24 hours, Patient returned with worsening symptoms, including headache, altered mental status, fever, and cough | - Patient had travel history returned from Europe 7 days earlier before the symptoms onset - Patient had history of atrial fibrillation, cardioembolic stroke, Parkinson’s Disease, COPD, and cellulitis - EEG shows diffuse slowing and focal slowing sharply contoured waves in the left temporal region indicate encephalopathy - Head CT-Scan showed no acute changes, other than the presence of hypodensity area indicates prior history of embolic stroke - the CSF findings showed no signs of infection | - Diagnostic test was limited | 5 |
| Korea Centers for Disease Control and Prevention,^33^ 2020) | | Case Series | South Korea | 28 patients with laboratoryconfirmed COVID-19. | Mean Age (y) 42.6 (20-73 y) | - Headache, 3 (10.7%) - Myalgia, 4 (14.3%) | - 18/28 (64.3%) patients are tested positive pneumonia by imaging | - Not specifically studied neurological symptoms - No clear definition of symptoms - Diagnostic test was limited | 4 |
| Zhao et al,^34^ 2020 | | Retrospective  Cohort | China | 101 patients with RT-PCR positive COVID-19 | 43 (17–75 y) | - Nausea and Vomiting, 2 (2%) - Myalgia, 17 (16.8%) | - 16/101 (15.8%) patients have cerebrovascular underlying diseases | - Not specifically studied neurological symptoms - No clear definition of symptoms - Diagnostic test was limited | 3 |
| Xu et al,^35^ 2020 | | Retrospective Cohort | China | 50 patients with RT-PCR positive COVID-19 | Mean Age (y) ± SD:  43.9 ±16.8 | - Headache, 5 (10%) - Myalgia, 8 (16%) | - Headache was mostly found in patients with moderate disease 3/50 (6%) - Myalgia was mostly found in patients with moderate disease 4/50 (8%) | - Not specifically studied neurological symptoms - No clear definition of symptoms - Diagnostic test was limited | 3 |
| Li et al,^36^ 2020 | | Retrospective Cohort | China | 131 patients with RT-PCR positive COVID-19 | Mean Age (y) ± SD:  47 ± 15 | - Myalgia, 2 (2%) | - Not available | - Not specifically studied neurological symptoms - No clear definition of symptoms - Diagnostic test was limited | 3 |
| Wang et al,^37^ 2020 | | Retrospective Cohort | China | 1012 patients with RT-PCR positive COVID-19 from sputum or nasoharyngeal swab | 50 (16-89 y) | - Headache, 152 (15%) - Myalgia, 170 (16.8%) - Vomiting, 30 (3.3%) | - Patients included in the study were non critically ill patients. - 100/1012 patients had aggravation of illness during follow up - headache (19% vs 14.6%), myalgia (17% vs 16.8%), and vomiting (6% vs 3.3%) were more common among patients with aggravation of illness during follow up | - Not specifically studied neurological symptoms - No clear definition of symptoms - Diagnostic test was limited | 3 |
